# Long-Term Outcomes of Transarterial Chemoembolization for Intrahepatic Cholangiocarcinoma: Overall Survival and Tumor Response

**DOI:** 10.7759/cureus.101629

**Published:** 2026-01-15

**Authors:** Thomas J Vogl, Anna Hillebrand, John Bielfeldt, Fabian Finkelmeier, Ursula Pession, Hamzah Adwan

**Affiliations:** 1 Clinic for Radiology and Nuclear Medicine, University Hospital Frankfurt, Goethe University Frankfurt, Frankfurt, DEU; 2 Department of Gastroenterology and Hepatology, University Hospital Frankfurt, Goethe University Frankfurt, Frankfurt, DEU; 3 Department of General, Visceral, Transplantation and Thoracic Surgery, University Hospital Frankfurt, Goethe University Frankfurt, Frankfurt, DEU

**Keywords:** interventional radiology, intrahepatic cholangiocarcinoma, minimally invasive tumor therapy, oncology, transarterial chemoembolization

## Abstract

Purpose

This study aims to determine the efficacy of transarterial chemoembolization (TACE) for patients with inoperable intrahepatic cholangiocarcinoma (iCCA) who do not respond to systemic therapy, focusing on overall survival (OS) and tumor response.

Methods

A total of 339 patients (188 males and 151 females) with a mean age of 59.2 ± 12.8 years were retrospectively evaluated in the study. The OS time was calculated using the Kaplan-Meier method. Tumor response was determined using the Response Evaluation Criteria in Solid Tumors (RECIST).

Results

The median OS time was 10 months (95% CI: 8.4-11.6) with 6-, 12-, 24-, and 36-month OS rates of 70.5%, 43.9%, 14.3%, and 6.5%, respectively. The evaluation of tumor response according to RECIST was possible in 203 patients. The tumor volume decreased from 240.83 ± 431.80 cm^3 ^to 184.31 ± 316.88 cm^3^. The rates of stable disease, partial response, complete response, and progressive disease were 84.73% (172/203), 7.88% (16/203), 0.49% (1/203), and 6.9% (14/203), respectively. The local tumor control rate was 93.1% (189/203).

Conclusions

In case of inoperable and systemic therapy-resistant iCCA, interventional therapy using TACE shows high local tumor control rates among evaluable patients and satisfactory OS for this high-tumor-burden cohort. TACE should be considered as an alternative treatment option and applied in suitable patients. However, these results are limited by the retrospective study design and incomplete RECIST data.

## Introduction

Cholangiocarcinoma (CCA) represents a heterogeneous class of highly aggressive epithelial tumors characterized by cholangiocyte differentiation [1-3]. Depending on its presumed origin, CCA can be subclassified as intrahepatic, perihilar, or distal CCA [3,4]. In the United States, the overall incidence of CCAs is 2.1 to 3.3 cases per 100,000 person-years, making them rare; however, geographic variation exists [1,5-7]. Intrahepatic CCA (iCCA), which accounts for 10-20% of primary liver malignancies, is the second most common primary hepatobiliary carcinoma, after hepatocellular carcinoma (HCC) [1,6-8].

CCA is slightly more common in men, and an increasing incidence of iCCA has been observed in the United States [5,9]. In its early stages, CCA is often asymptomatic and is therefore often diagnosed at an advanced stage, when resectability is often compromised, and metastases may have developed. Due to the late onset of symptoms, CCA poses a significant health risk, with a low five-year survival rate [5,6]. While most cases of CCA occur sporadically and without a recognizable cause, certain risk factors have been identified, including primary sclerosing cholangitis (PSC), liver fluke infection (i.e., Opisthorchis viverrini), hepatolithiasis, biliary malformation (bile duct cysts, Caroli disease), and liver cirrhosis [6,10]. Depending on the stage, different therapy options are available for CCA. In cases of technically resectable tumors without distant metastases, the indication for possible curative liver resection is given [11-14]. However, contraindications such as cirrhosis, invasion of the portal vein, or distant metastases must be ruled out [15-17]. If resection cannot be performed, systemic therapy represents another treatment option [14].

According to the European Society for Medical Oncology (ESMO) guidelines, a combination of gemcitabine and cisplatin, supplemented by the immune checkpoint inhibitor durvalumab, is considered first-line treatment for patients with locally advanced, unresectable, or metastatic CCA [18]. In earlier studies, a combination of chemotherapy and durvalumab resulted in a survival time of 12.9 months, compared with 11.3 months for chemotherapy alone [19]. The median overall survival (OS) time for iCCA was 53 months after R0 resection in combination with capecitabine [20]. In this context, R0 resection means that no tumor can be detected at the resection margins after resection [21]. Alternatively, locoregional therapies, such as radiofrequency ablation (RFA), transarterial chemoembolization (TACE), transarterial radioembolization (TARE), brachytherapy, or microwave ablation (MWA), may also be considered in inoperable patients with CCA smaller than 3 cm [13,18]. During TACE, the tumor-feeding vessels are accessed to locally apply chemotherapeutic and embolic agents to achieve blood stasis and ensure high local concentrations of chemotherapeutic agents [22,23]. After TACE, the median survival time is between 12 and 17 months. In combination with systemic therapy, survival times extended by an additional 2-12 months [24]. TARE with yttrium-90 achieved a median survival of 12.7 months and a mean tumor volume reduction of 35% [25,26]. A combination of TARE and systemic therapy achieved survival times of 22 months [27]. Therefore, this study aims to investigate the long-term efficacy of TACE for patients with inoperable and systemic therapy-resistant iCCA.

## Materials and methods

Ethics committee approval

The ethical committee of the university hospital has approved this single-center study. Informed consent was waived due to the retrospective nature of the study.

Patients data

This retrospective study encompasses individuals diagnosed with inoperable and not systemic therapy-responding iCCA, who were treated between January 2000 and September 2022 at our clinic for radiology. The patient cohort consists of 339 patients, all of whom were treated with TACE as monotherapy. The collected patient data was pseudonymized. All patients were treated by the same experienced interventional radiologist throughout the entire observation period.

Selection criteria

The inclusion criteria were as follows: (1) iCCA as a primary tumor, (2) treatment by TACE alone, and (3) preserved hepatic function. The exclusion criteria were as follows: (1) presence of multiple primary tumors, (2) poor general condition, (3) portal vein thrombosis, and (4) coagulopathy.

TACE

During TACE, a catheter was inserted into the femoral artery via the Seldinger technique for subsequent angiographic visualization of the vessels. The angiographic findings were utilized to evaluate the arterial supply of the tumor. Following, a standard triple of chemotherapeutic agents and embolic agents was introduced via the catheter to obstruct the tumor’s blood supply and impede its growth. The employed chemotherapeutic agents included mitomycin C, irinotecan, cisplatin, and gemcitabine. In case of allergy, doxorubicin was used in the treatment alternatively. Chemotherapeutics were selected after discussing the patients’ cases in a multidisciplinary tumor board. Additionally, embolization agents such as Lipiodol and degradable starch microspheres were applied. The successful devascularization was confirmed by angiography following the procedure.

Follow-up protocol

A CT scan was performed after TACE mainly to detect possible complications. For evaluation of response to TACE, contrast-enhanced MRI was performed. Thereafter, imaging follow-up examinations were performed at three-month intervals for the first year, followed by examinations at six-month intervals.

Data analysis

The raw data from the clinic's proprietary database were processed to evaluate the following parameters: age, gender, date of first and last intervention, number of interventions, current status (deceased, alive), OS time, volume, and diameter before and after the TACE procedure. Furthermore, the cytostatic drugs utilized for TACE were identified. A picture archiving and communication system (PACS) was used for image analysis. Hereby, tumor sizes were determined based on cross-sectional imaging. If sufficient data were available, response to treatments was utilized according to the Response Evaluation Criteria in Solid Tumors (RECIST 1.0) [28]. Patients were also classified according to local tumor control. The RECIST classifications, stable disease (SD), complete response (CR), and partial response (PR), are counted as local tumor control. The evaluation according to RECIST criteria was not feasible in all patients due to a lack of follow-up imaging. Additionally, OS was calculated from the date of initial treatment to the date of last contact or death.

Statistical methods

The statistical analysis of this study was conducted using IBM SPSS Statistics for Windows, Version 30 (Released 2024; IBM Corp., Armonk, New York, United States). The OS was calculated using the Kaplan-Meier method. Nominal variables were presented as frequencies and percentages, and continuous variables as mean and standard deviation.

## Results

Over a period of more than 22 years, a total of 339 patients diagnosed with iCCA were treated at our institute. The cohort consisted of 188 males and 151 females with a mean age of 59.2 ± 12.8 years. The iCCA patients were treated with TACE alone. The total number of TACE procedures performed on all patients was 1739, with a mean of 5.13 procedures per patient. Table 1 shows an overview of the general characteristics of the patients.

**Table 1:** Baseline characteristics of patients and treatment details. TACE: transarterial chemoembolization

Parameters	Value
Male, n (%)	188 (55.5)
Female, n (%)	151 (44.5)
Age mean ± standard deviation in years	59.2 ± 12.8
Performed treatments in total n	1739
Mean number of TACE procedures per patient	5.13

Tumor response

The mean tumor volume was 240.83 ± 431.80 cm^3^ prior to the initial TACE intervention and reduced to 184.31 ± 316.88 cm^3^ following the final intervention, resulting in an average tumor volume reduction of 23.47%. The mean tumor diameter prior to the initial TACE was 7.27 ± 4.41 cm. After the last TACE, the mean tumor diameter reduced to 6.78 ± 4.30 cm. These diameters and volumetric measurements illustrate an overall reduction in tumor burden achieved over the course of the interventions, and can be seen in Table 2.

**Table 2:** Tumor volume and diameter changes in patients undergoing TACE. TACE: transarterial chemoembolization

Parameters	Value
Average tumor volume prior to TACE ± standard deviation in cm^3^	240.83 ± 431.80
Average tumor volume after last TACE ± standard deviation in cm^3^	184.31 ± 316.88
Mean tumor diameter prior to TACE in cm ± standard deviation in cm	7.27 ± 4.41
Mean tumor diameter after last TACE in cm ± standard deviation in cm	6.78 ± 4.30

**Figure 1:**
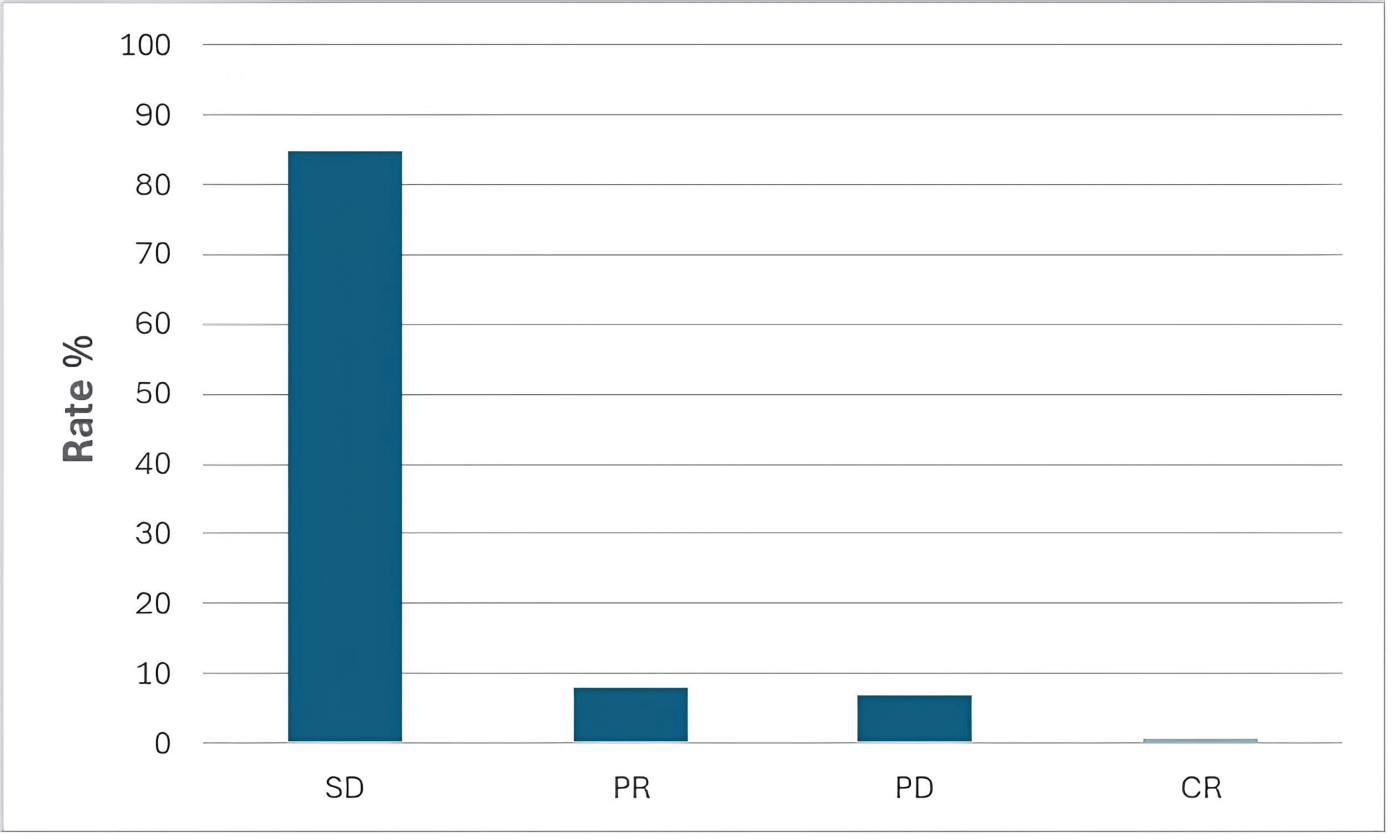
Tumor response according to the RECIST. SD: stable disease; PR: partial response; PD: progressive disease; CR: complete response; RECIST: Response Evaluation Criteria in Solid Tumors

The evaluation of tumor response according to RECIST was possible in 203 patients. It was not possible to assess tumor response in the remaining 136 patients due to missing data, in particular, radiological imaging. During therapy, most patients achieved an SD at 84.73% (172/203). PR was observed in 7.88% (16/203) of patients, and progressive disease in 6.9% (14/203) of patients. CR could be achieved in 0.49% (1/203) of patients. The local tumor control rate was 93.1% (189/203). Figure 1 shows a bar chart of the tumor response according to RECIST.

Overall survival (OS)

**Figure 2:**
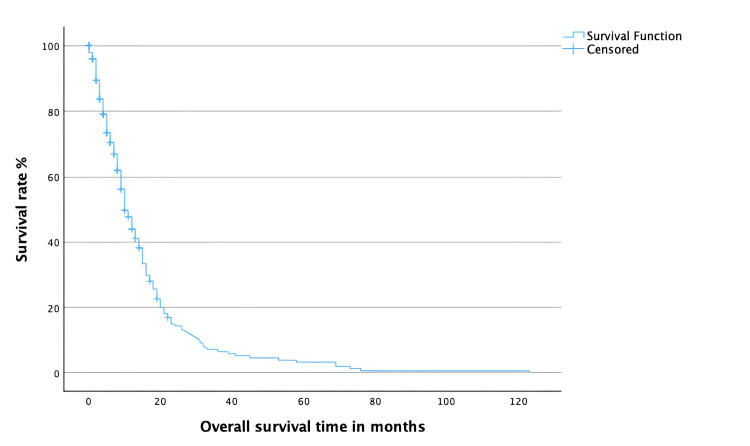
Kaplan-Meier curve for overall survival.

The 6-, 12-, 24-, and 36-month OS rates were 70.5%, 43.9%, 14.3%, and 6.5%, respectively. The median OS was 10 months (95% CI: 8.4-11.6), while the mean OS was 15.2 months (95% CI: 12.8-17.5). A total of 133 out of 339 patients (39.2%) were still alive at the last follow-up and were therefore censored. Figure 2 shows the OS curve.

## Discussion

CCA is a malignancy with an unfavorable prognosis and high mortality rate [2,5]. Survival times for iCCA are usually less than 24 months after diagnosis [2]. Surgical resection is considered the best therapeutic option; however, it is only feasible for a limited number of patients (approximately 30-35%) due to contraindications such as invasion of the main portal vein [2,12,13,15]. According to Moris et al., recurrences occur in most patients after curative resection of iCCA, which are often multifocal and extrahepatic and are therefore no longer suitable for further resection and must be treated with alternative modalities [20]. In patients with unresectable tumors, chemotherapy is administered [22,29]. Consequently, there is a need for alternative treatment options, and advances in locoregional therapies are becoming increasingly relevant in the management of CCA [22].

This study aimed to contribute to the growing body of evidence regarding the role of locoregional therapies in the treatment of iCCA, with a particular focus on TACE as a monotherapy. Overall, the results of this study were meant to demonstrate the therapeutic efficacy of TACE monotherapy in patients with unresectable and systemic therapy non-responsive high-burden iCCA.

In this study, the mean tumor reduction after TACE was 23.47%, with a tumor control rate of 93.1% in evaluable patients. These outcomes are higher than comparable results of previously reported studies, such as an objective response rate of 20.6% after TACE alone in unresectable iCCA in a study by Mosconi et al. [30].

Treatment efficacy, however, is known to be influenced by several variables such as initial tumor size, lesion count, and liver function. This was supported by a meta-analysis by Kim et al. that showed that patients with tumors larger than 3 cm, those with multiple tumors, and patients older than 65 years exhibited a less favorable outcome after thermal ablation procedures [31]. Since the average diameter of the tumors treated in this study was greater than 3 cm before the first intervention, the less favorable outcomes described by Kim et al. were to be expected. Based on this information, the median survival rate of 10 months, which is poor compared to other TACE studies (12-17 months), appears plausible and contextually justified [24]. Despite the lower median survival, TACE showed considerable efficacy in local tumor control, aligning with prior findings in similar cohorts. The current ESMO guidelines recommend gemcitabine and cisplatin in combination with durvalumab for systemic chemotherapy [18]. However, other chemotherapeutic drugs, including irinotecan and mitomycin C, were also employed in this study.

Ray et al. performed a meta-analysis including 16 articles comprising a total of 542 patients and reported a one-year OS of 58% for TACE-treated patients, with a survival benefit of two to seven months compared to systemic chemotherapy [32]. Although TACE demonstrates a survival benefit, surgical resection and liver transplantation remain the treatment modalities associated with the most favorable long-term outcomes. Moris et al. reported a median survival of 53 months with R0 resection and adjuvant therapy with capecitabine [20]. Lang et al. found a median survival of 25.8 months with resection. The three-year survival rates were 39% [33], compared with 6.5% for TACE in our study. The relatively low three-year-survival rate may be explained by the fairly high initial tumor burden. In addition, the comparison shows that resection is still superior. According to the meta-analysis by Ziogas et al., liver transplantation is associated with a markedly prolonged survival period, with a one-year survival rate of 75% and a three-year survival rate of 56% [34]. However, CCA remains a contraindication for transplantation in most centers [34], and surgical resection is frequently not an option due to advanced disease or the presence of metastases [13]. Neoadjuvant systemic therapy and locoregional therapies should be employed in cases of locally advanced disease or large lesions (>2 cm) [14,35]. These approaches may increase the likelihood of secondary curative resection over the course of therapy [35]. Table 3 offers a comparative overview of survival outcomes reported for various iCCA treatment modalities in the literature, including the results from our cohort. It also provides a clear comparison of survival times under different therapies. Hereby, R0 resection clearly provides the best results.

**Table 3:** Comparative overview of published treatment modalities and associated outcomes for iCCA. TACE: transarterial chemoembolization; iCCA: intrahepatic cholangiocarcinoma; TARE: transarterial radioembolization; OS: overall survival

Therapy	Median OS in months	Three-year survival (%)
R0 resection	25.8 [33]	39 [33]
Systemic therapy	12.9 [19]	14.6 [36]
TARE	12.7 [25]	16.8 [37]
TACE (current study)	10	6.5

This study has several limitations that must be considered when interpreting the results. Firstly, we must acknowledge that this is a retrospective study, potentially prone to selection bias. Furthermore, the lack of randomization may have affected the validity of the results, and the absence of a control group (e.g., patients receiving systemic therapy alone or best supportive care) limits the ability to directly compare treatment efficacy with other alternative treatment options. Although we attempted to contextualize our results through literature comparisons (as shown in Table 3), prospective controlled studies are necessary to confirm these findings. Furthermore, we could not evaluate tumor response according to RECIST in all patients due to a lack of follow-up imaging. Despite the valuable long-term results of this study, it must be acknowledged that treatment protocols, including the chemotherapeutic drugs utilized, have changed over the course of two decades, which in turn leads to therapeutic heterogeneity that has a potential impact on these results. A retrospective standardization of the treatment protocols was not possible and represents another relevant limitation of this study. Henceforth, future studies with longer observational periods should systematically record and acknowledge changes in treatment protocols. Despite these limitations, the present analysis reflects the actual clinical development of treatment procedures over the observed long-term period. Lastly, this study did not evaluate treatment tolerability, adverse events, or quality of life. However, these aspects are of essential importance to conclusively assess the clinical-therapeutic relevance of the respective procedures. Without this information, the holistic assessment of the benefits and burden of the respective therapies on patients remains incomplete.

## Conclusions

This study included patients with high-burden iCCA who were inoperable and did not respond to systemic therapies. Our long-term results showed that TACE, a minimally invasive interventional treatment, may provide a high local tumor control rate among the evaluable patients and satisfactory OS for this high-tumor-burden cohort. Therefore, TACE may be a relevant and effective treatment option for applicable patients. However, further studies are necessary to verify these results and potentially compare TACE with other treatment options to further improve patient outcomes.
